# Ultrabroadband Tellurium Photoelectric Detector from Visible to Millimeter Wave

**DOI:** 10.1002/advs.202103873

**Published:** 2021-12-19

**Authors:** Wanli Ma, Yanqing Gao, Liyan Shang, Wei Zhou, Niangjuan Yao, Lin Jiang, Qinxi Qiu, Jingbo Li, Yi Shi, Zhigao Hu, Zhiming Huang

**Affiliations:** ^1^ State Key Laboratory of Infrared Physics Shanghai Institute of Technical Physics Chinese Academy of Sciences 500 Yu Tian Road Shanghai 200083 P. R. China; ^2^ University of Chinese Academy of Sciences 19 Yu Quan Road Beijing 100049 P. R. China; ^3^ Technical Center for Multifunctional Magneto‐Optical Spectroscopy (Shanghai) Engineering Research Center of Nanophotonics & Advanced Instrument (Ministry of Education) Department of Materials School of Physics and Electronic Science East China Normal University 500 Dongchuan Road Shanghai 200241 P. R. China; ^4^ Donghua University 2999 North Renmin Road Shanghai 201620 P. R. China; ^5^ Key Laboratory of Space Active Opto‐Electronics Technology Shanghai Institute of Technical Physics Chinese Academy of Sciences 500 Yu Tian Road Shanghai 200083 P. R. China; ^6^ Hangzhou Institute for Advanced Study University of Chinese Academy of Sciences 1 Sub‐Lane Xiangshan Hangzhou 310024 P. R. China; ^7^ Institute of Optoelectronics Fudan University 2005 Songhu Road Shanghai 200438 P. R. China

**Keywords:** electromagnetic induced wells, photodetection, tellurium, THz imaging, ultrabroadband

## Abstract

Ultrabroadband photodetection is of great significance in numerous cutting‐edge technologies including imaging, communications, and medicine. However, since photon detectors are selective in wavelength and thermal detectors are slow in response, developing high performance and ultrabroadband photodetectors is extremely difficult. Herein, one demonstrates an ultrabroadband photoelectric detector covering visible, infrared, terahertz, and millimeter wave simultaneously based on single metal–Te–metal structure. Through the two kinds of photoelectric effect synergy of photoexcited electron–hole pairs and electromagnetic induced well effect, the detector achieves the responsivities of 0.793 A W^−1^ at 635 nm, 9.38 A W^−1^ at 1550 nm, 9.83 A W^−1^ at 0.305 THz, 24.8 A W^−1^ at 0.250 THz, 87.8 A W^−1^ at 0.172 THz, and 986 A W^−1^ at 0.022 THz, respectively. It also exhibits excellent polarization detection with a dichroic ratio of 468. The excellent performance of the detector is further verified by high‐resolution imaging experiments. Finally, the high stability of the detector is tested by long‐term deposition in air and high‐temperature aging. The strategy provides a recipe to achieve ultrabroadband photodetection with high sensitivity and fast response utilizing full photoelectric effect.

## Graphical abstract

In this work, ultrabroadband photoelectric detectors covering visible, infrared, terahertz, and millimeter wave simultaneously are demonstrated through two kinds photoelectric effect synergy of photoexcited electron–hole pairs and electromagnetic induced well effect based on metal–Te–metal structure. The strategy of utilizing full photoelectric effect provides an effective route to achieve ultrabroadband photodetection with high sensitivity and fast response.

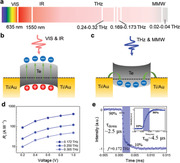

## 1 Introduction

Photodetector can convert light into electrical signal, which is the core to obtain information. At present, photodetection technology is developing toward high sensitivity and broadband response. High‐performance broadband photodetection has great significance in a variety of cutting‐edge technologies including imaging, communications, and medicine.^[^ ^1^ ^]^ For example, a broadband photodetector can quickly obtain spectral information of different bands, which significantly improves the identification of the target. In addition, broadband photodetector can be used for wavelength‐selective photodetector applications.^[^ ^2^ ^]^

However, covering visible (VIS), infrared (IR), terahertz (THz), and millimeter wave (MMW) bands simultaneously poses a significant challenge for high‐performance broadband photodetection. Conventional photodetection based on photoexcited electron–hole pairs in a semiconductor is effective to detect VIS and IR. But it does not perform well for long‐wavelength, especially for the THz and MMW ranges due to the energy of the photons lower than the bandgap and strong background fluctuation.^[^ ^3^ ^]^ Commercial long‐wavelength photodetectors currently consist primarily of quantum well photodetectors (QWPs), field‐effect transistors (FETs), and Schottky barrier diodes (SBDs).^[^ ^4^ ^]^ However, they often require complex material growth and/or fabrication techniques, and QWPs operate only at low temperatures. In addition, thermal detectors generally suffer from a slow response.^[^ ^5^ ^]^ Therefore, it is extremely urgent to explore new strategies for realizing high‐sensitivity broadband photodetection.

Recently, the detectors with metal–semiconductor–metal (MSM) structure were proposed based on narrow gap semiconductors HgCdTe (MCT), in which when an electromagnetic wave with photon energy much less than the bandgap of the MCT impinges on the MSM structure, an electromagnetic induced well (EIW) is generated in the semiconductor, it traps the carriers from the metal, then the conductivity of the semiconductor is changed and the photovoltage signal of electromagnetic wave could be detected.^[^ ^6^ ^]^ In recent years, with the deep research on the micro–nano machining technology, unprecedented attention has been paid to 2D materials.^[^ ^7^ ^]^ At present, vast 2D materials including graphene, black phosphorus, transition‐metal dichalcogenides (TMDs), and topological materials, have been confirmed to be used for photodetection.^[^ ^8^ ^]^ Due to many unique advantages of 2D materials, the replacement of MCT with 2D materials is of great significance to fast response and broadband photodetection.

Tellurium (Te) is an important p‐type narrow‐bandgap semiconductor with a bandgap of ≈0.35 eV.^[^ ^9^ ^]^ Te belongs to Group VI, and has a unique chiral‐chain crystal lattice.^[^ ^10^ ^]^ While a mass of synthetic elemental 2D materials is generally unstable in ambient air, Te exhibits excellent air stability, high mobility, and other desirable properties, including photoconductivity, thermoelectricity, and piezoelectricity, making it an attractive material for sensors, optoelectronics, and energy devices.^[^ ^9^, ^10^, ^11^ ^]^ At present, there have been a large number of reports on photoelectric devices based on Te and its heterojunction, such as transistors and IR detectors.^[^ ^12^ ^]^ Te FETs exhibit on/off ratios on the order of 10^6^, and field‐effect mobilities of about 700 cm^2^ V^−1^ s^−1^.^[^ ^13^ ^]^ The Te nanowires IR photodetectors exhibit a responsivity of 6650 A W^−1^ and a detectivity of 1.23 × 10^12^ Jones (at 1550 nm).^[^ ^14^ ^]^ However, there is no report about the Te detectors in long wavelength.

In this work, we successfully fabricated Te‐based room temperature full photoelectric detectors with high‐sensitivity and ultrabroadband covering VIS, IR, THz, and MMW simultaneously, due to the synergy of photoexcited electron–hole pairs and EIW effect.^[^ ^6^ ^]^ The detector does not require multiple material combinations with complex structures, instead, it relies on a single semiconductor material–Te to address the issue of broadband detection. Furthermore, high‐resolution THz images obtained at room temperature demonstrate the detector's superior performance and potential applications. Finally, the high stability of the detector is tested by long‐term deposition in air and high‐temperature aging. Our strategy provides an effective route to achieve ultrabroadband photodetection with high sensitivity and fast response utilizing the full photoelectric effect.

## 2 Results and Discussion

**Figure 1:**
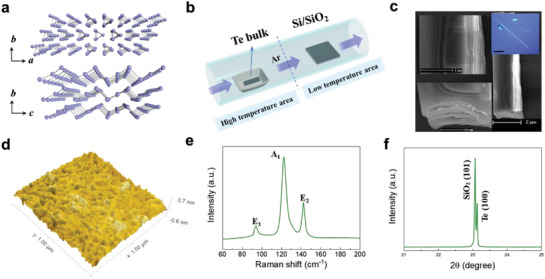
Material characteristics of Te nanosheets. a) Schematic diagram of the crystal structure. b) Schematic of PVD growth of Te nanosheets. c) SEM images of Te nanosheets. Inset: Optical image of Te nanosheet (Scale: 100 µm). d) AFM scan of Te nanosheet showing excellent uniformity. e) Raman spectrum of Te nanosheet. f) XRD pattern of Te nanosheet on the Si/SiO_2_ substrate.

Te crystal has a unique chiral‐chain crystal lattice in which individual helical chains of Te atoms are stacked together by van der Waals type bonds and each Te atom is covalently bonded with its two nearest neighbors on the same chain as shown in **Figure** **1**a.^[^ ^10^ ^]^ Herein, to explore the performance of Te nanosheets in photodetection, the high‐quality Te nanosheets were grown by physical vapor deposition (PVD). A Te bulk as the precursor is placed in a quartz boat in the high‐temperature area, and the inverted SiO_2_/Si substrate is in the low‐temperature area as shown in Figure 1b. The details of material growth are described in the Experimental Section. The microstructure and surface morphology of the Te nanosheets were measured by scanning electron microscope (SEM) and atomic force microscopy (AFM) in Figure 1c,d. The inset is the optical image of Te nanosheets grown on Si/SiO_2_ substrate. The layered structures are clearly shown in the SEM images. The AFM image shows smooth sample surface. The arithmetic average roughness *R*_a_ is about 0.08387 nm, and the root‐mean‐squared roughness *R*_rms_ is about 0.1102 nm. To further analyze the quality and crystal structure of the Te nanosheets, we conducted the Raman and the X‐ray diffraction (XRD) measurements. The Raman spectra of the Te nanosheets are displayed in Figure 1e. The Te nanosheets exhibited three main Raman‐active modes located at about 93, 121, and 141 cm^−1^, which represent E_1_, A_1_, and E_2_ modes, respectively.^[^ ^15^ ^]^ The phase and purity of Te nanosheets were inspected by the XRD. The XRD pattern is shown in Figure 1g. From Figure 1g, we can see that Te nanosheets are single‐crystalline with growth direction along (100), and the diffraction peaks can be indexed as the hexagonal crystal structure (JCPDS file no. 36‐1452).^[^ ^16^ ^]^ All of them imply the formation of high‐quality Te nanosheets which are significant to fabricating high‐performance detectors.

**Figure 2:**
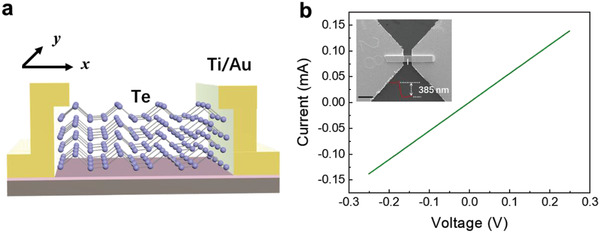
The structure and electrical characteristics of the Te detector. a) Schematic diagram of Te detector structure. b) The *I–V* characteristic of the detector. Inset: a SEM image of a detector (Scale: 20 µm).

Through semiconductor fabrication technology such as ultraviolet lithography, dual ion beam technique, lift‐off, spot welding, etc., we fabricated the MSM structure (**Figure** **2**a) of the Te detector, which can work at room temperature. The detailed fabricating processes are shown in the Experimental Section and Figure S1 (Supporting Information). The *I–V* characteristic of the detector was measured from ‐0.25 to 0.25 V and excellent ohmic behavior was observed (Figure 2b). The inset is the SEM image of a detector. The height was measured by AFM as shown in Figure S2 (Supporting Information). The channel length of the detector is 10 µm, the width of the Te nanosheet is about 8 µm, and the thickness is about 385 nm.

**Figure 3:**
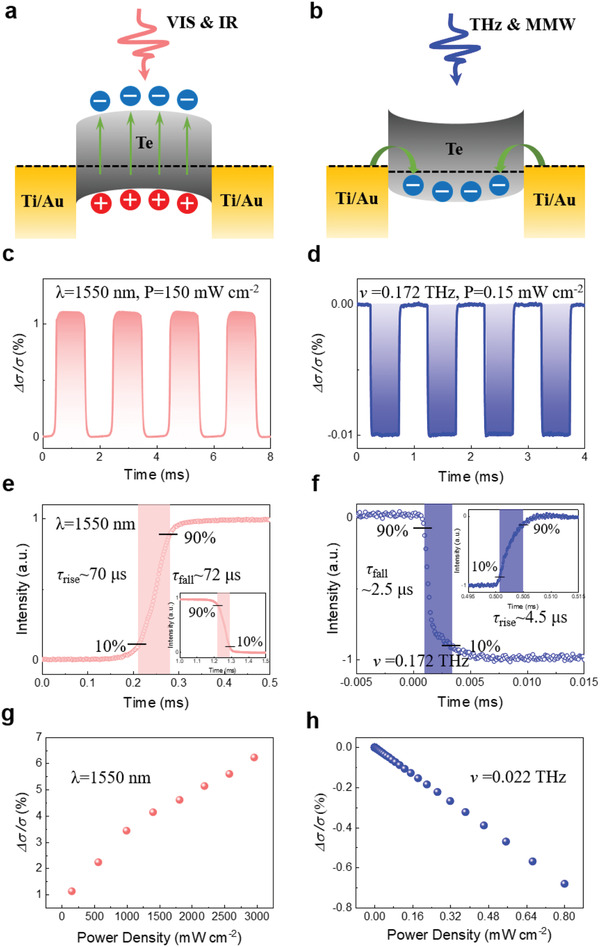
The detection mechanism of the Te detector in different bands. a) Band structure of Ti/Au–Te–Ti/Au under VIS and IR radiation. b) Band structure of Ti/Au–Te–Ti/Au under THz and MMW radiation. c,d) Positive photoconductivity on IR (1550 nm) radiation and negative photoconductance on THz (0.172 THz) radiation with a bias voltage 0.2 V, respectively. e,f) Response time on IR (1550 nm) and THz (0.172 THz) radiation. g,h) Change in conductance as changing the power of 1550 nm laser and 0.022 THz radiation with a bias voltage 0.2 V.

Next, we explored the performance of Te detectors. Broadband detection based on Te is caused by photoexcited electron–hole pairs and EIW effect. In the VIS and IR bands, due to the energy of the incident light exceeding the bandgap of the Te (*hν* ≥ *E*_g_), nonequilibrium electron–hole pairs are formed as shown in **Figure** **3**a. And under the applied electric field, the photogenerated carriers are respectively collected by the anode and cathode electrodes. What is more, the Te detector can directly detect THz and MMW. The photon energy of THz and MMW are much less than the Te bandgap of 0.3 eV, the extra carriers cannot be photo‐generated by interband transitions. Nevertheless, according to previous research on EIW, the antisymmetric electric field of THz and MMW radiation can be utilized in the MSM structure and an electromagnetic‐induced well will be formed when the detector is irradiated. In the one‐half period when the antisymmetric electric field appears parallel to the surface of the detector, the electrons in the metallic electrodes are emitted into the semiconductor Te by Lorentz force (Figure 3b). In the following half period, the injected electrons are decelerated but further move forward the center along the trajectory. ^[^ ^6^ ^]^ Then electrons recombine with holes in the p‐type semiconductor Te, which changes the resistance of the Te nanosheet. Therefore, positive photoconductivity was observed in the VIS and IR bands, while negative photoconductance was observed in the THz and MMW bands as shown in Figure 3c,d. In addition, the resistance change of the detector was measured in the temperature range of 200–325 K. It can be seen from Figure S3 (Supporting Information) that as the temperature increases, the resistance of the detector becomes larger in the temperature range of 200–250 K, and the resistance decreases in the temperature range of 250–325 K. And the temperature coefficient of resistance (TCR) goes from positive to negative as described in the report.^[^ ^17^ ^]^ Therefore, under the THz and MMW radiation, the temperature of the detector will rise slightly and the resistance will decrease. However, it was observed that under the THz and MMW radiation, the resistance of the detector increases, which is the opposite of the result caused by the thermal effect. In addition, the thermal response speed is relatively slow, generally on the order of ≈ms,^[^ ^5^ ^]^ which is much longer than the response time (≈2.5 µs) of the detector in THz and MMW bands. As a consequence, the contribution from the thermal effect can be excluded.

Furthermore, the photothermoelectric effect usually requires an asymmetric structure in the device or a light source that irradiates unevenly the device.^[^ ^18^ ^]^ But our detector is a symmetrical MSM structure, the light source is uniformly illuminated on the surface, and there is no photoresponse without bias voltage in Figure S4 (Supporting Information). In order to observe the positive and negative photoconductivity more intuitively, we measured the *I–V* of a detector at dark, MMW and IR as shown in Figure S4 (Supporting Information). When the detector is illuminated by IR, the current in the circuit increases; however, when the detector is illuminated by MMW, the current decreases. It can be seen that the long‐wavelength photoresponse is a photoconductivity phenomenon that is different from the photoexcited electron–hole pairs caused. And the photothermoelectric effect can be excluded.

Moreover, the response time which is usually defined as the time measured from 10% up to 90% on the rising edge of signal as well as the recovery time (from 90% down to 10% of the falling edge), under 1550 nm laser radiation, the response time *τ*_rise_ and *τ*_fall_ of the detector are 70 and 72 µs as shown in Figure 3e; under THz radiation, the response time *τ*_rise_ and *τ*_fall_ are 4.5 µs and 2.5 µs as shown in Figure 3f. It is because the speed of the light response caused by light excitation mainly depends on the radiation lifetime, while in the EIW effect, the response speed is dominated by the Auger lifetime of carriers.^[^ ^19^ ^]^ To verify the different response characteristics of the detector, the relative conductance Δ*σ/σ* as a function of powers at 1550 nm and 0.022 THz was measured at room temperature with a bias voltage 0.2 V as shown in Figure 3g,h.

Moreover, to verify the performance of the detector in the VIS and IR region, the photocurrent and responsivity of the detector with a bias voltage 0.2 V at 1550 nm are shown in Figure S5 (Supporting Information). The photocurrent under different bias voltages from 0 to 0.5 V at 635 nm were measured at room temperature (Figure S6a, Supporting Information). The responsivities reach 0.793 A W^−1^ and 9.38 A W^−1^ under the illumination of 635 nm and 1550 nm lasers at a bias voltage of 0.2 V, respectively. We recorded the waveforms of the detector at 635 nm in Figure S6b (Supporting Information). The response time *τ*_rise_ and *τ*_fall_ are 44.5 µs at 635 nm as shown in Figure S6c,d (Supporting Information). It is less than the response time at 1550 nm, which is consistent with the reported results.^[^ ^14^ ^]^

**Figure 4:**
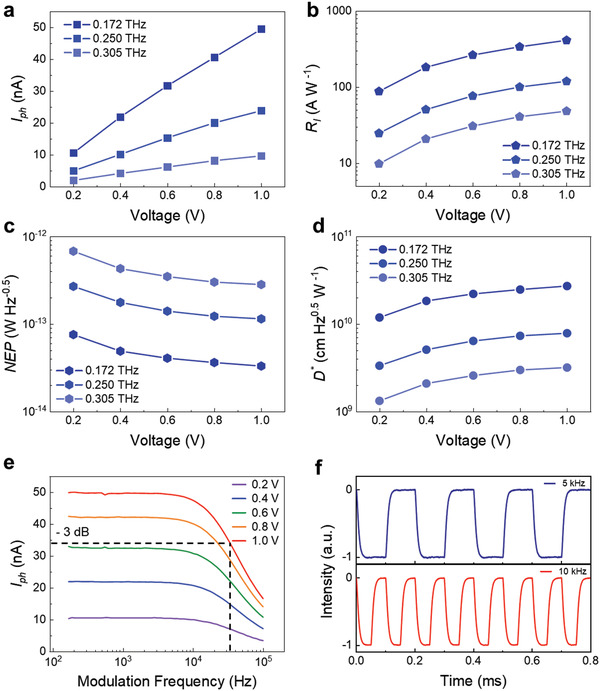
The performance of the Te detector in the THz band. a–d) The *I*_ph_, *R*_I_, NEP, and D^*^ with the bias voltage from 0.2 to 1.0 V at 0.172 , 0.250, and 0.305 THz. e) Amplitude–frequency response of the detector from 0.2 to 1.0 V. f) The waveforms of the detector at 5 and 10 kHz.

In order to further verify the performance of the Te detector in the THz and MMW bands, we measured the response in the range of 0.24–0.32, 0.169–0.173, and 0.02–0.04 THz at room temperature as shown in Figure S7a–c (Supporting Information). The setup of the THz and MMW test system is presented in Figure S8 (Supporting Information). The photocurrent increases linearly with the increase of the bias voltage at 0.172, 0.250, and 0.305 THz in **Figure** **4**a. The responsivity (*R*_I_), noise equivalent power (NEP), and detectivity (D^*^) are important indicators to measure the performance of the detector. Herein, the responsivity is defined as $R_{I}=\frac{I_{\mathrm{ph}}}{P\times A}\;$, *I*_ph_ is the photocurrent, *P* is the power density of the source radiated to the surface of the Te detector, *A* is the photoactive area of the detector.

According to the EIW effect, the concentration of the trapped electrons Δ*n* in the well can be expressed as: ^[^ ^6^ ^]^

(1) $$\begin{array}{ccc} \Delta n & = & \frac{4\varepsilon_{0}a\eta P}{\pi^{3}q^{2}dc_{0}\sqrt{\varepsilon_{r}}}\;\sqrt{{\left(\frac{\pi }{a}\right)}^{2}-k_{0}^{2}}\\ & & \times \;\left[1-\exp\left(-d\sqrt{\varepsilon_{r}}\sqrt{{\left(\frac{\pi }{a}\right)}^{2}-k_{0}^{2}}\right)\right] \end{array}$$

where *a* is the channel length of the device, *d* is the thickness of the nanosheet, $k_{0}=\frac{2\pi \nu }{c_{0}}\;$ is the free space wave vector, *ν* is the frequency of light. Correspondingly, the responsivity can be expressed as: ^[^ ^6^ ^]^

(2) $$\begin{array}{ccc} R_{I} & = & \frac{4\varepsilon_{0}a\eta V_{b}\Gamma_{e}}{\pi^{3}q^{2}drnc_{0}\sqrt{\varepsilon_{r}}}\;\sqrt{{\left(\frac{\pi }{a}\right)}^{2}-k_{0}^{2}}\\ & & \times \;\left[1-\exp\left(-d\sqrt{\varepsilon_{r}}\sqrt{{\left(\frac{\pi }{a}\right)}^{2}-k_{0}^{2}}\right)\right] \end{array}$$

The relationship between photocurrent *I*_ph_ and Δ*n* can be expressed as: $I_{\mathrm{ph}}=\frac{\Delta n}{n}\;\cdot I_{b}$, and $I_{b}=\frac{V_{b}}{r}\;$ *, V*_b_ is the bias voltage, *r* is the resistance of the device. Therefore, according to the expectation of EIW theory, it can be seen that *I*_ph_∝ *P* (Figure S9, Supporting Information), *I*_ph_∝ *V*_b_ (Figure 4a), *R*_I_∝ *V*_b_ (Figure 4b) and *R*_I_ is constant as the power changes (Figure S10, Supporting Information). Affected by the item${\left(\frac{\pi }{a}\right)}^{2}-k_{0}^{2}$, as the frequency of incident light increases, the responsivity decreases rapidly (Figure S11, Supporting Information). Therefore, these experiments not only show that the Te detector has extremely excellent performance, but also verify the EIW theory.

NEP is the lowest detectable power per unit of bandwidth. NEP can be expressed as $\mathrm{NEP}\;=\frac{i_{n}}{R_{I}}\;$, *i*_n_ is the root mean square of the noise current. The total noise can be described by $i_{n}={\left(\frac{4k_{B}T}{r}+2eI_{b}\right)}^{0.5}\;$,^[^ ^3^ ^]^ *k*_B_ is the Boltzmann constant, *r* is the resistance of the device, *e* is the unit charge, and *I*_b_ is the dark current, here it is the bias current. The detectivity D^*^ is the main parameter for characterizing the normalized signal‐to‐noise performance of detectors and can be defined as $D^{*}=\frac{A^{0.5}}{\mathrm{NEP}}\;$. Figure 4b–d shows the measured *R*_I_, NEP, and D^*^ of the Te detector at different voltage bias for room temperature operation, where the detector exhibits excellent performance. According to calculation, under a bias voltage of 0.2 V, our detector has a *R*_I_ of up to 87.8 A W^−1^, a NEP of 7.58 × 10^−14^ W Hz^‐0.5^ and a D^*^ of 1.19 × 10^10^ cm Hz^0.5^ W^−1^ in THz band. In addition, the photocurrent was recorded with the modulation frequency *f* from 150 Hz to 100 kHz at 0.172 THz. The detector demonstrated a 3 dB bandwidth of 3.4 × 10^4^ Hz, corresponding to a response time of 4.68 µs according to $\tau \;=\frac{1}{2\pi f_{-3\;\mathrm{dB}}}\;$,^[^ ^5^ ^]^ which is consistent with the measurement result of the pulse in Figure 3f. We recorded the waveforms of the detector at 5 and 10 kHz in Figure 4f. This shows that our detector is much faster than commercial Golay, pyroelectric, and uncooled semiconducting microbolometer detectors (ms level). It can still meet the requirement of real‐time imaging applications. All in all, the Te detector has shown outstanding detection performance. The performance in each band at a bias of 0.2 V is shown in Table S1 (Supporting Information).

**Figure 5:**
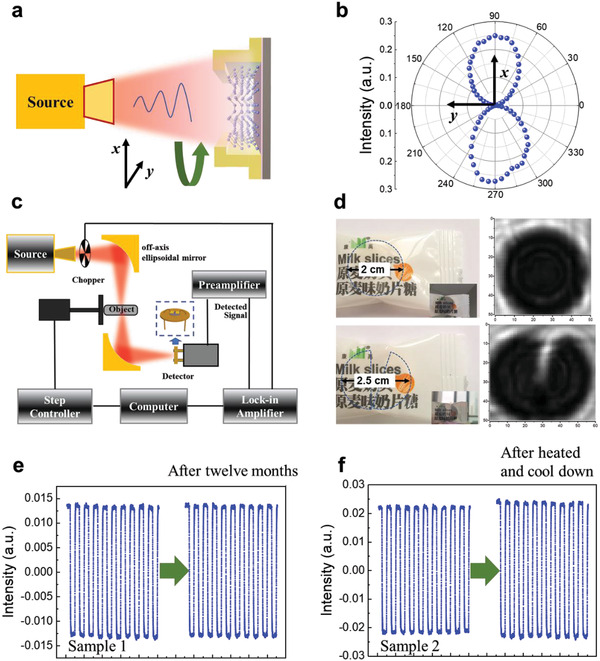
The imaging and stability of Te detector for potential application. a) Schematic diagram of polarization measurement. b) Polarization properties of Te detector at 0.022 THz. c) Schematic of the THz transmission scanning imaging. d) The high‐resolution images were obtained by the setup. e) The response of the detector was monitored before and after deposited in the atmosphere for twelve months. f) The detector was heated to 200 °C kept for 20 min, and the measured response remained almost the same after the detector cooled down to room temperature.

In addition, the polarization dependence of the response at 0.022 THz was measured. The schematic diagram of polarization detection is shown in **Figure** **5**a. The response is the largest when the polarization is along *x* axis (TM), decreases as the polarization deviates from the *x* axis, and finally disappears when the polarization is along *y* axis (TE), which ultimately results in the lobe‐like polarization diagram, as shown in Figure 5b. Due to the EIW formed inside the semiconductor, the polarization effect of the antenna (Figure S12, Supporting Information) and excellent anisotropy of the Te crystal,^[^ ^14^ ^]^ the detector exhibits good polarization detection with a polarization extinction ratio of 468. In addition, we compared the polarization extinction ratio of Te and other 2D materials under different wavelengths/frequencies of light as shown in Table S2 (Supporting Information).^[^ ^14^, ^20^ ^]^

In order to verify the application of the detector, THz transmission scanning imaging experiments were performed. The THz source was tuned to 0.305 THz and operated at room temperature under an ambient environment. Schematic of the setup is shown in Figure 5c. The two milk tablets in the plastic package were hidden as the test objects. In Figure 5d, it can be seen through THz imaging experiments that one of the milk tablets in the opaque plastic package was intact and the other was cracked. This shows that the Te detector can be used in rapid imaging applications, such as identifying hidden objects and food quality inspection.

The high stability of the detector is an important prerequisite for its wide application. Lots of synthetic elemental 2D materials are generally unstable in the ambient atmosphere, but Te exhibits good stability. In this work, the robustness of the Te detectors to ambient atmosphere and heat treatment was evaluated. The detectors without encapsulation were placed in the atmosphere for a long time, and the performance at 0.022 THz was monitored before and after the treatments, which has hardly changed as shown in Figure 5e. In order to further verify the stability, the detectors were heated to 200 °C for 20 min. After the detector cooled down, the response remained almost the same, as shown in Figure 5f. In addition, we measured the Raman spectra of Te nanosheet that was placed for more than 12 months, then heated at 200° for 20 min, the three main Raman‐active modes did not change significantly as shown in Figure S13 (Supporting Information). Therefore, the Te nanosheets and the detectors exhibit high stability under various harsh working environments.

## 3 Conclusions

In summary, we have developed an ultrabroadband full photoelectric detector based on Te semiconductor with excellent sensitivity and high stability covering VIS, IR, THz, and MMW simultaneously. The response of the detector in the VIS and IR bands originates from the photoexcited electron–hole pairs. The responsivity is 0.793 A W^−1^ at 635 nm and 9.38 A W^−1^ at 1550 nm. Based on the EIW effect, the detector also has an excellent response in the THz and MMW bands with the responsivities of 9.83 A W^−1^ at 0.305 THz, 24.8 A W^−1^ at 0.250 THz and 87.8 A W^−1^ at 0.172 THz and a dichroic ratio of 468 at 0.022 THz. Due to the different photoelectric detection mechanisms, the detector has a fast response (≈4.5 µs) in the THz band, compared with the response in the VIS (≈44.5 µs) and IR (≈70 µs) bands. THz performed imaging experiments have proved that our detector can be used to distinguish hidden objects. Moreover, the high stability of the Te detector has been verified by long‐term deposition in air and high temperature aging experiments. Our results show that Te is an ideal material for broadband photoelectric detection, and the synergy of photoexcited electron–hole pairs and the EIW effect has important theoretical guiding significance in broadening the response band and improving the response speed of photodetectors.

## 4 Experimental Section

### Grown of Te Nanosheets

The Te nanosheets were grown by a PVD method. The Te bulk as a prelude was placed into the quartz boat, then this quartz boat was placed in the high‐temperature reaction area located in the tube furnace cavity; the inverted Si/SiO_2_ as a substrate was placed on the quartz boat, then this quartz boat was placed in the low‐temperature growth area. After vacuuming, argon was pumped in and the residual air in the quartz tube was discharged. During the reaction, argon was continuously flowed in at a flow rate of 40 sccm. In the two temperature areas, it took 45 min for the high‐temperature zone to 450 °C, and the low‐temperature zone to 280 °C. The temperature was maintained for 30 min in the two temperature areas. At last, it dropped to room temperature and the Te nanosheets had been grown on the substrate. The thickness and size of Te nanosheets can be well controlled by adjusting the growth conditions.

### Detector Fabrication

First, the photoresist AZ4330 was spin‐coated on the substrate with Te nanosheets. Then the samples were put in a dryer for drying. The electrode and antenna patterns were prepared by ultraviolet lithography (SUSS MJB4) and development (AZ400K). Then the samples were put into the chamber of the dual ion beam sputtering system, and sputtered Ti and Au, respectively, with thicknesses of 30 and 300 nm. After peeling off in acetone solution, the samples were held on the base by resin epoxy glue. Finally, the electrodes of detectors and the pins of base were connected with gold wires through a spotwelding technology.

### Characterization and Measurements

Raman spectra were performed on confocal microscopy (HR 800) under the 532 nm laser. Electronic and optoelectronic properties of Te detectors were performed at room temperature under ambient conditions. The data of response time were acquired from an oscilloscope (Teledyne LeCroy 62Xi‐A). For VIS and IR detection, the power of 635 nm laser is 3 mW, and the spot diameter is about 1 mm; the power of 1550 nm laser is continuously adjustable, and the spot diameter is about 5 mm. For THz and MMW detection, the electronic frequency multiplication source (Virginia Diodes Inc.), a backward wave tube (Microtech Instruments Inc.) and MMW signal generator (Agilent E8257D) were modulated as the sources. After the detected signals were amplified by the preamplifier (SR 570), they were read by the lock‐in amplifier (SR 830), which modulated the signal source at the same time. Then the detector was replaced by a Golay cell to record the incident power of THz and MMW under the exact same measurement conditions. The average power density irradiated on the surface of the detector at 0.022 THz, 0.172 THz, 0.250 THz and 0.305 THz is about 0.8, 0.15, 0.25, 0.25 mW cm^−2^, respectively.

### Statistical Analysis

The photocurrent was measured through the preamplifier and lock‐in amplifier. The responsivity is defined as $R_{I}=\frac{I_{\mathrm{ph}}}{P\times A}\;$, where *I*_ph_ is the photocurrent, *P* is the power density of the source radiated to the surface of the Te detector, *A* is the photoactive area of the detector. NEP is calculated by formulas $\mathrm{NEP}\;=\frac{i_{n}}{R_{I}}\;$, where $i_{n}={\left(\frac{4k_{B}T}{r}+2eI_{b}\right)}^{0.5}\;$ is the root mean square of the noise current, *k*_B_ is the Boltzmann constant, *r* is the resistance of the device, *e* is the unit charge, and *I*_b_ is the dark current, here it is the bias current. The detectivity D^*^ can be defined as $D^{*}=\frac{A^{0.5}}{\mathrm{NEP}}\;$. The software used to organize and analyze experimental data is Excel and Origin. The software used to simulate the gain of the antenna is HFSS.

## Conflict of Interest

The authors declare no conflict of interest.

## Supporting information

Supporting Information

## Data Availability

Research data are not shared.
