# Supplementary material for: Ultrabroadband Tellurium Photoelectric Detector from Visible to Millimeter Wave
Source: Adv Sci (Weinh). 2021 Dec 19;9(5):2103873. doi: 10.1002/advs.202103873 (PMC8844568; doi:10.1002/advs.202103873)
Supplement: Supplementary file 1 — Supporting Information [file ADVS-9-2103873-s001.pdf]

ADVANCED SCIENCE
Open Access

Supporting Information

for *Adv. Sci.*, DOI: 10.1002/advs.202103873

Ultra-Broad Band Tellurium Photoelectric Detector from Visible to Millimeter Wave

Qiu, *Jingbo Li*, *Yi Shi*, *Zhigao Hu*, and *Zhiming Huang*[*]

Supporting Information

Ultra-Broad Band Tellurium Photoelectric Detector from Visible to Millimeter Wave

*Wanli Ma, Yanqing Gao**, *Liyan Shang, Wei Zhou, Niangjuan Yao, Lin Jiang, Qinxi Qiu, Jingbo Li, Yi Shi, Zhigao Hu, Zhiming Huang**

W. Ma, Associate Prof. Y. Gao, Associate Prof. L. Shang, Associate Prof. W. Zhou, N. Yao, L. Jiang, Q. Qiu, J. Li, Prof. Z. Huang
State Key Laboratory of Infrared Physics
Shanghai Institute of Technical Physics
Chinese Academy of Sciences
500 Yu Tian Road, Shanghai 200083, P. R. China
E-mail: zmhuang@mail.sitp.ac.cn
Prof. Z. Huang
Key Laboratory of Space Active Opto-Electronics Technology
Shanghai Institute of Technical Physics
Chinese Academy of Sciences
500 Yu Tian Road, Shanghai 200083, P. R. China
Prof. Z. Huang
Hangzhou Institute for Advanced Study
University of Chinese Academy of Sciences
1 Sub-Lane Xiangshan, Hangzhou 310024, P. R. China
Prof. Z. Huang
Institute of Optoelectronics, Fudan University
2005 Songhu Road, Shanghai 200438, P. R. China
W. Ma, Q. Qiu, J. Li
University of Chinese Academy of Sciences
19 Yu Quan Road, Beijing 100049, P. R. China
Associate Prof. L. Shang, Prof. Z. Hu
Technical Center for Multifunctional Magneto-Optical Spectroscopy (Shanghai), Engineering Research Center of Nanophotonics & Advanced Instrument (Ministry of Education), Department of Materials, School of Physics and Electronic Science, East China Normal University, Shanghai 200241, P. R. China
Y. Shi
Donghua University, 2999 North Renmin Road, Shanghai 201620, P. R. China

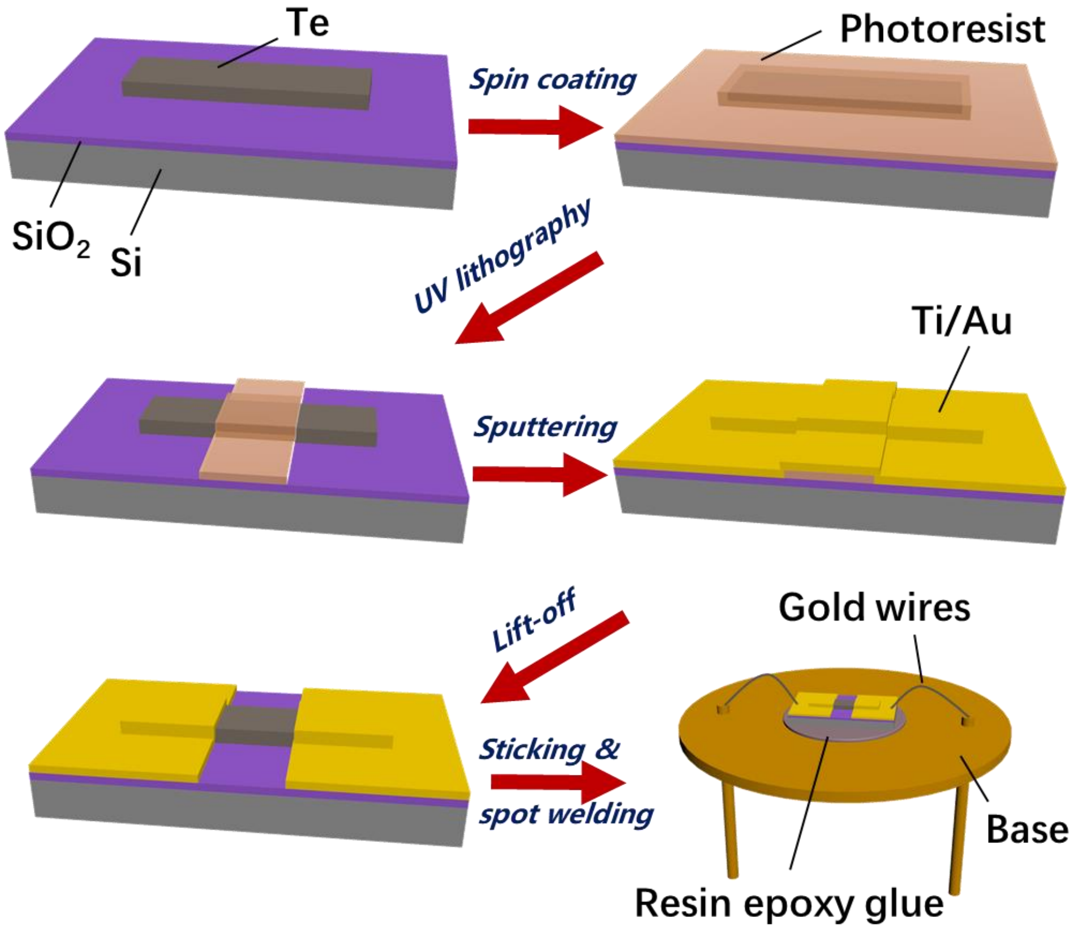

Figure S1. The fabrication process of the Te detector.

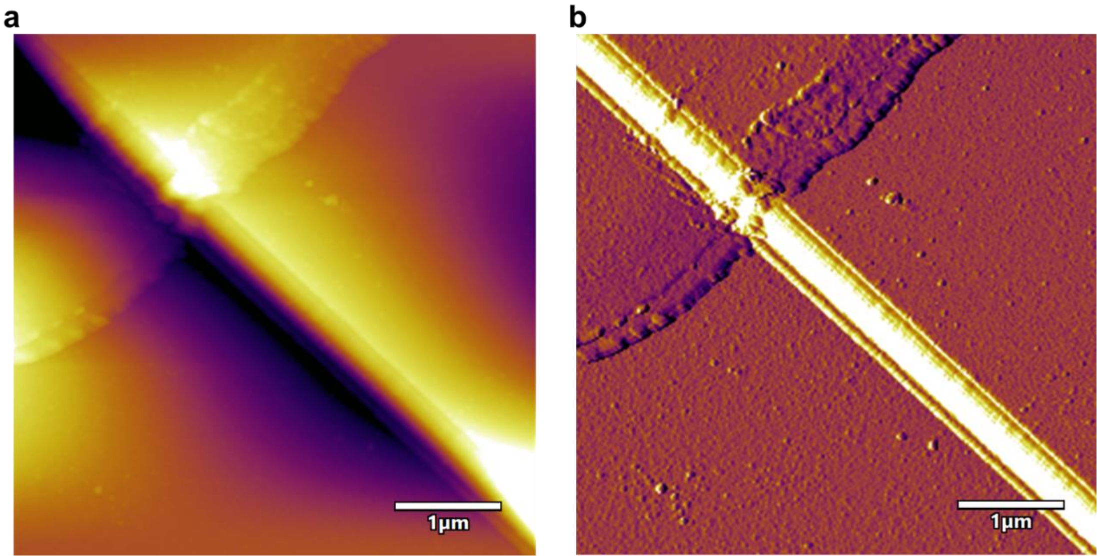

Figure S2. The height image **a** and amplitude image **b** of a Te detector measured by AFM.

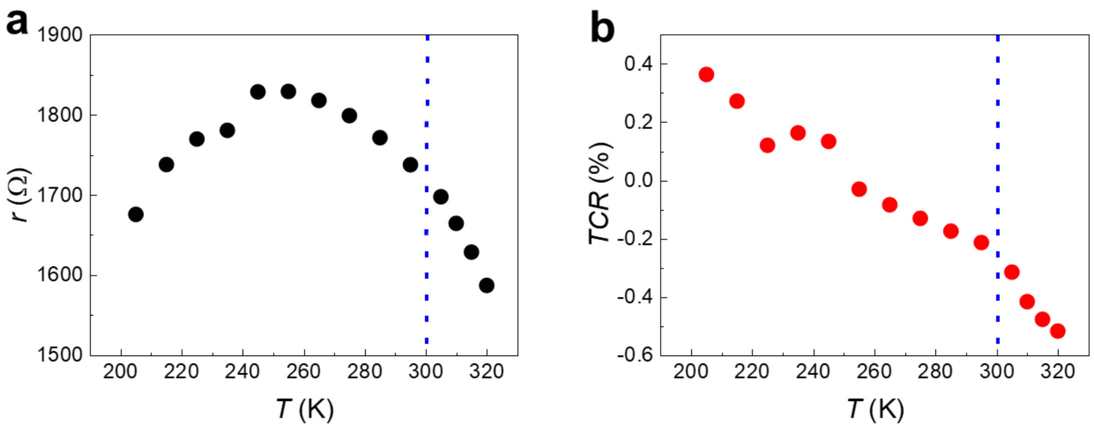

Figure S3. Variable temperature electrical characteristics of detector. The resistance **a** and *TCR* **b** change of the Te detector in the temperature range of 200-320 K.

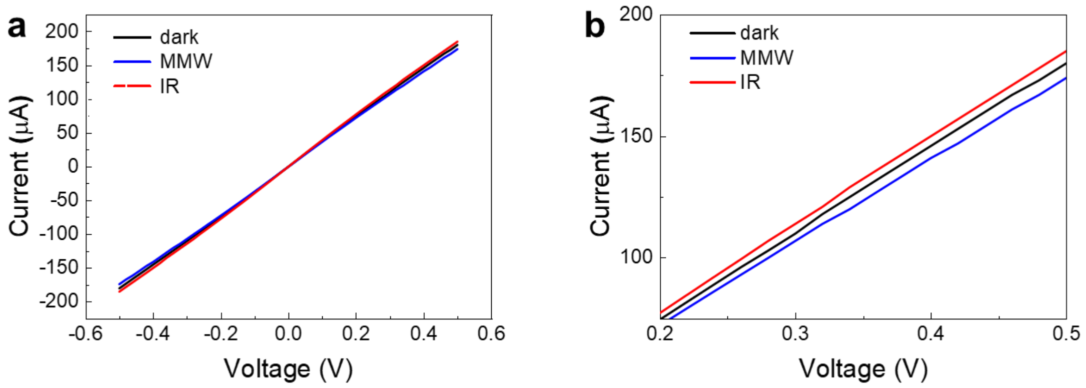

Figure S4. a) *I-V* of the device at dark, MMW radiation and IR radiation from -0.5 V to 0.5 V. b) *I-V* of the device at dark, MMW and IR from 0.2 V to 0.5 V.

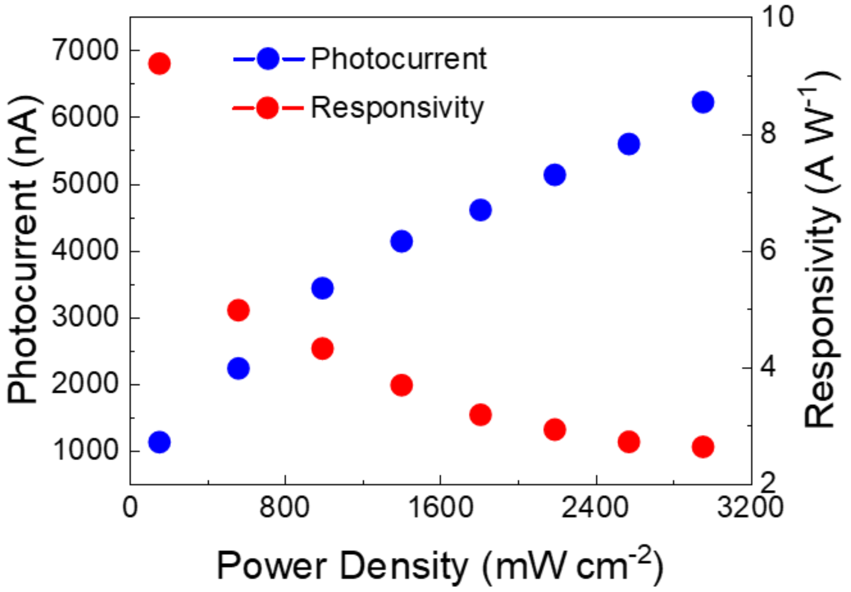

Figure S5. The photocurrent and responsivity of Te detector with a bias voltage 0.2 V at 1550 nm.

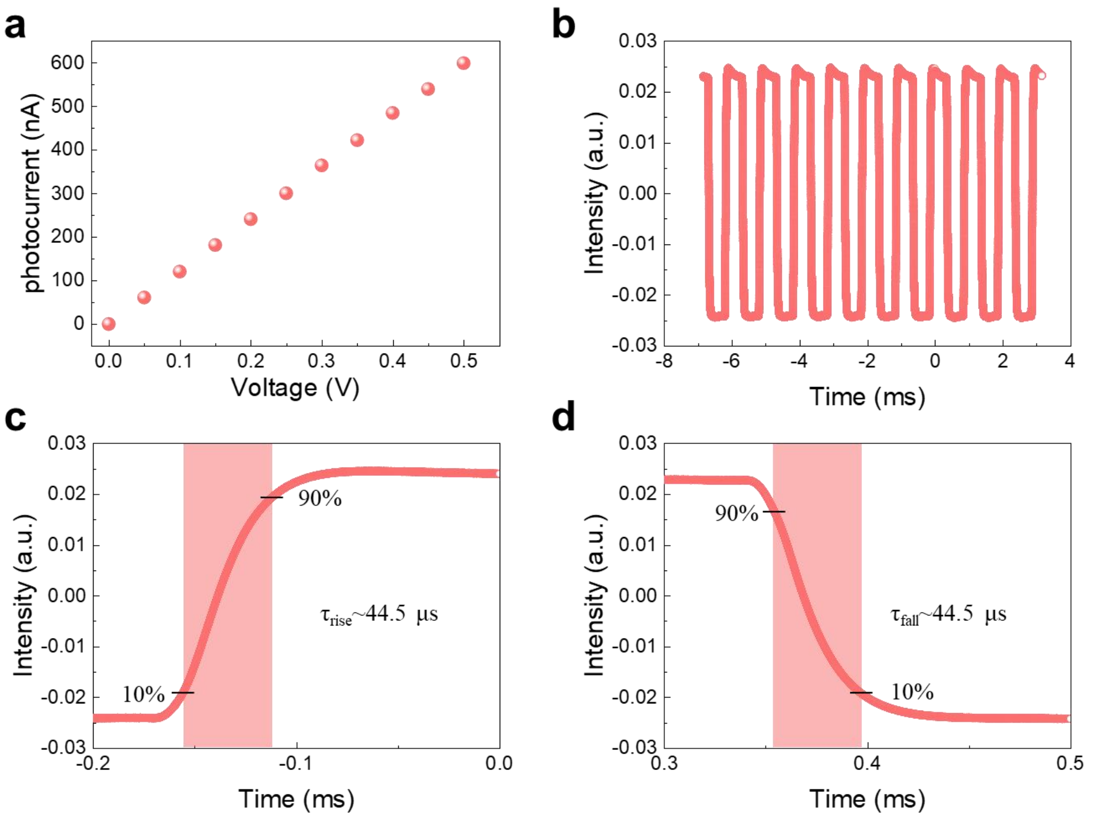

Figure S6. The performance of the Te detector at 635 nm. a The photocurrent under different bias voltages from 0 to 0.5 V at 635 nm. **b** The waveforms of the Te detector with a modulation frequency 1000 Hz at 635 nm. **c,d** Response time τ_{rise} and τ_{fall} are approximately 44.5 μs at 635 nm.

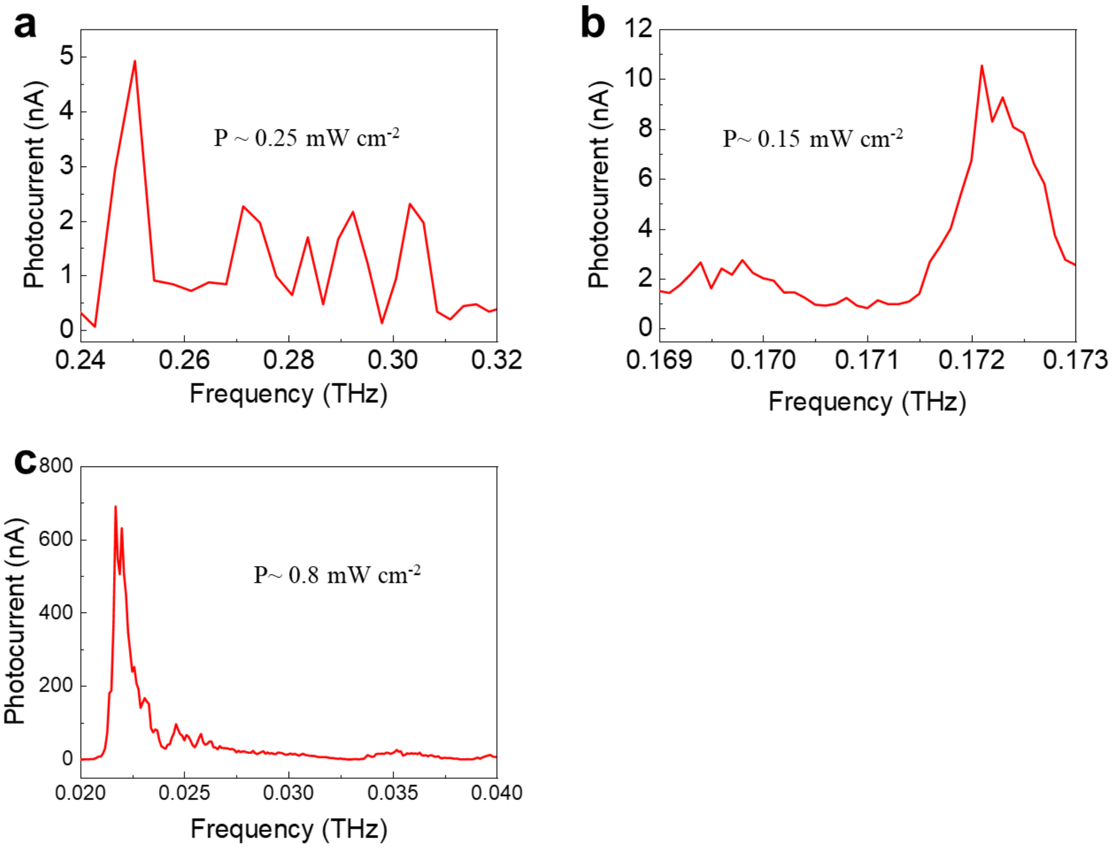

Figure S7. The photocurrent of Te detector in the ranges of 0.24-0.32 THz, 0.169-0.173 THz and 0.02-0.04 THz at room temperature.

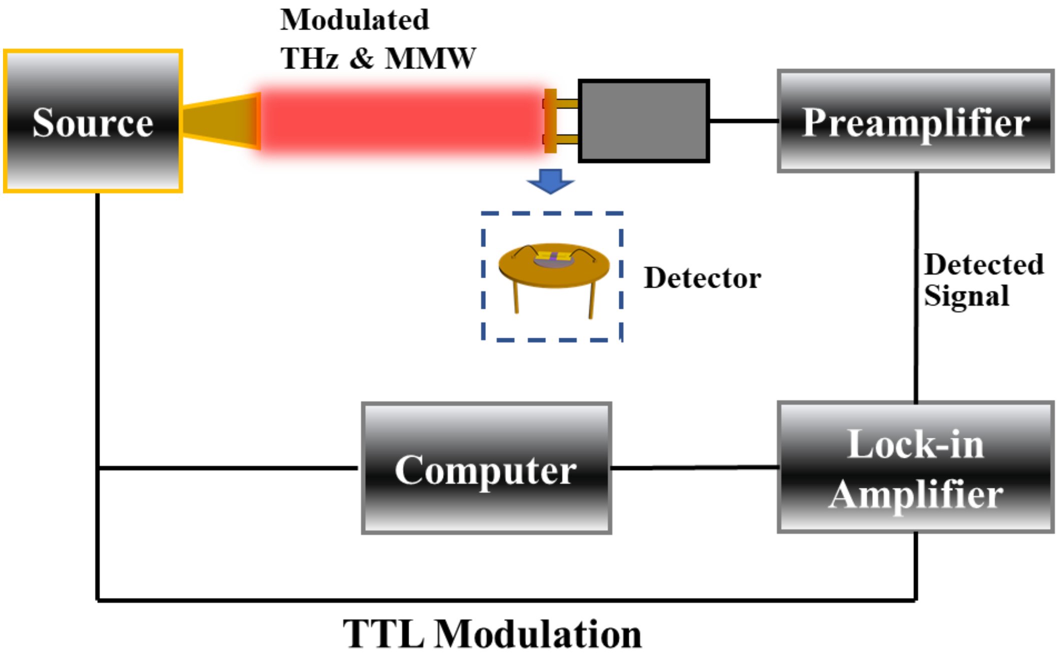

Figure S8. The setup of the THz and MMW measurement system. During the measurements, the modulated THz and MMW are vertically incident to the surface of the detector.

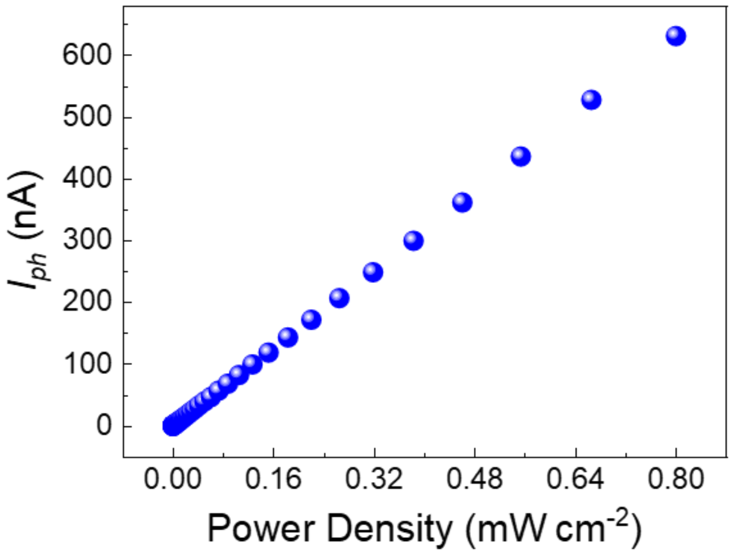

Figure S9. Photocurrent I_{ph} as changing the power of MMW radiation (0.022 THz) at 0.2 V.

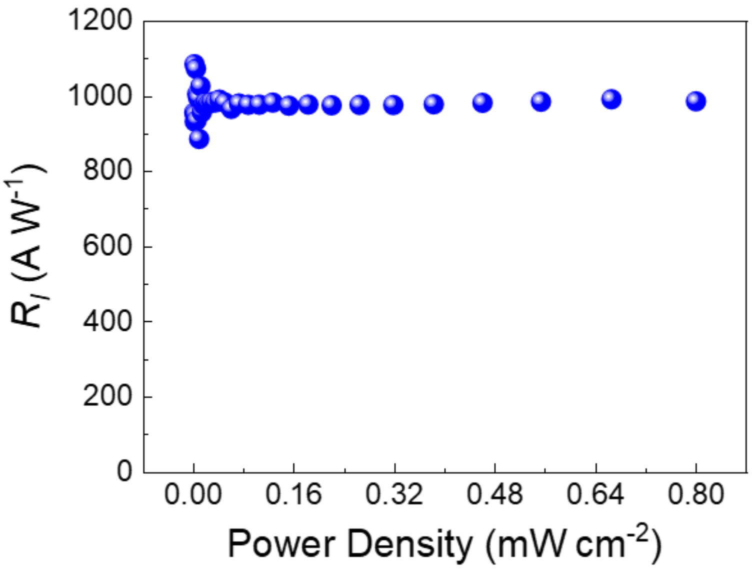

Figure S10. Responsivity R_I as changing the power of MMW radiation (0.022 THz) at 0.2 V.

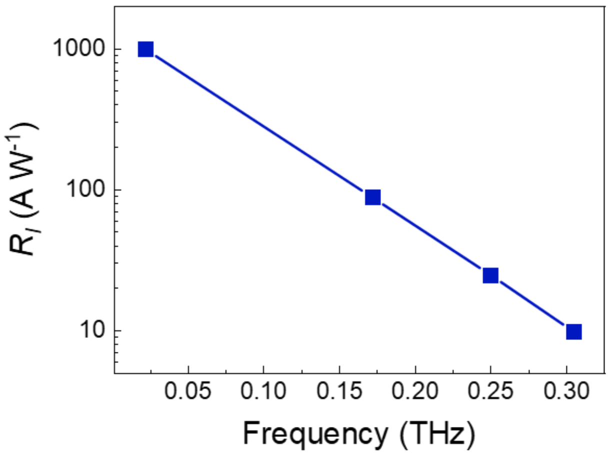

Figure S11. Responsivity R_I at 0.022 THz, 0.172 THz, 0.250 THz and 0.305 THz with the bias voltage 0.2 V.

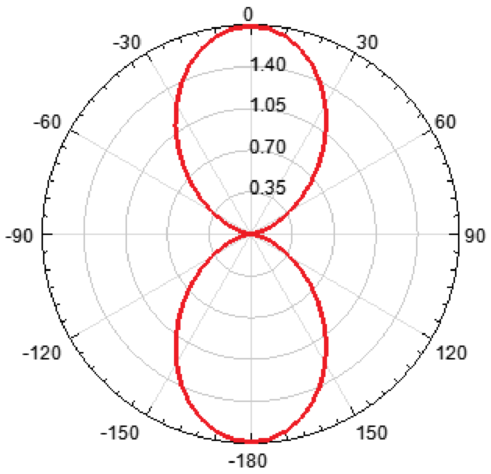

Figure S12. The relationship between the gain of the antenna and the azimuthal angle.

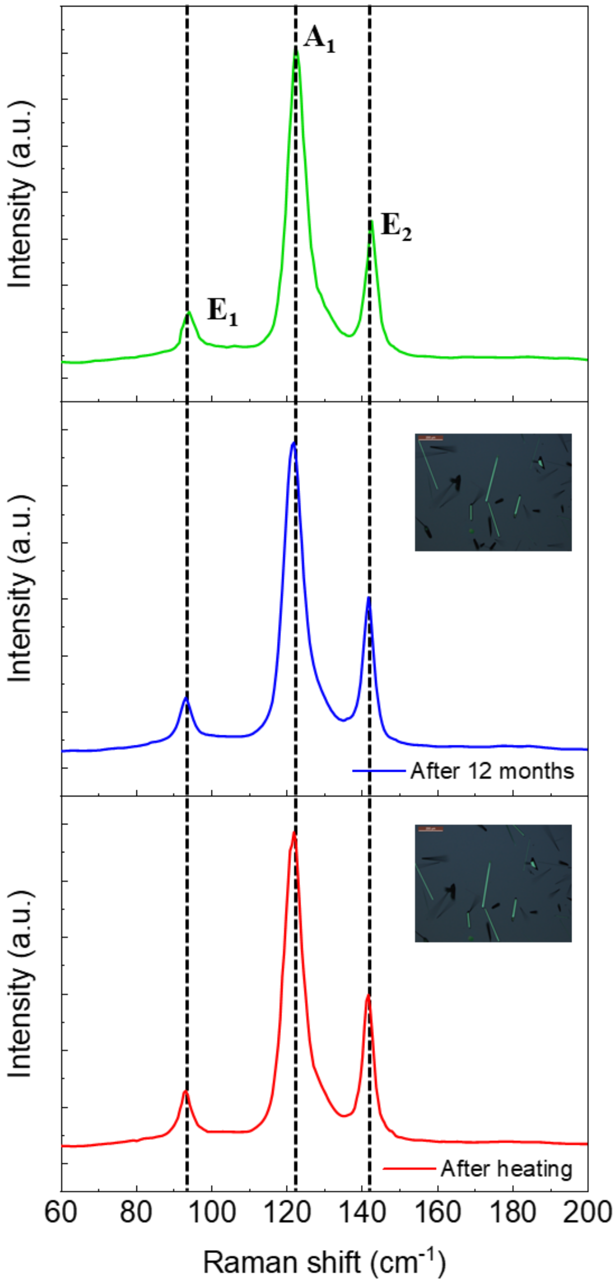

Figure S13. The Raman spectra of the device after 12 months and heating. The illustrations are optical pictures of the samples. Scale: 200 μm.

Table S1. The performance of the Te detector in each band at a bias of 0.2 V.

λ /*f*		I_{ph} [nA]	*P* [mW cm^{-2}]	T_{rise} / τ_{fall} [µs]	R_I [A W^{-1}]	*NEP* [W Hz$^{-0.5}$]	D^* [cm Hz$^{0.5}$ W^{-1}]
VIS	635 nm	241	380	44.5/44.5	0.793	8.39×10^{-12}	1.07×10^{8}
IR	1550 nm	1126	150	70/72	9.38	7.10×10^{-13}	1.27×10^{9}
THz	0.305 THz	1.97	0.25	4.5/2.5	9.83	6.78×10^{-13}	1.33×10^{9}
	0.250 THz	4.96	0.25		24.8	2.69×10^{-13}	3.35×10^{9}
	0.172 THz	10.6	0.15		87.8	7.58×10^{-14}	1.19×10^{10}
MMW	0.022 THz	631	0.8		986	6.74×10^{-15}	1.34×10^{11}

Table S2. Comparison with previous report of Te and other 2D materials in polarization dependence.

Material	Wavelength (nm) /Frequency (THz)	Polarization extinction ratio	References
Te	830 nm	5.8	[14]
	blackbody	2.4	
Te	2300 nm	~7.58	[20a]
	520 nm	~2.55	
	637 nm	2.66	
	785 nm	2.04	
	1550 nm	~2.39	
Te	1500 nm	~1.5	[20b]
	3000 nm	~10	
black phosphorus	500 nm	~4.5	[20c]
	1200 nm	3.5	
black phosphorus	0.29 THz	120	[20d]
Bi_2O_2Se	0.17 THz	110	[20e]
Te	0.022 THz	468	This work
